# Supplementary material for: Divergent effects of short-term and continuous anthropogenic noise exposure on Western Bluebird parental care behavior
Source: PeerJ. 2024 Nov 26;12:e18558. doi: 10.7717/peerj.18558 (PMC11606327; doi:10.7717/peerj.18558)
Supplement: Supplemental Information 1 [file peerj-12-18558-s001.docx]

**Table S1**. Model parameter estimates from top-ranked model in Table 6 for failed provisioning attempts in short-term noise exposure trials, minus activity from one influential Western Bluebird female.

| **Fixed Effects** | **Estimate** | **Std. Error** | **95% CI*** |
| --- | --- | --- | --- |
| (Intercept) | -24.4638 | 2.8888 | -30.10, -19.80 |
| Treatment Noise | 21.3249 | 2.8888 | 15.70, 26.99 |
| Brood size | 0.4824 | 0.387 | -0.28, 1.24 |
| Parent male | -0.3463 | 0.5386 | -1.40, 0.71 |

*Calculated using estimate ± (SE*1.96) due to convergence issues when calculating 95% CIs using confint() function in R.
