# Supplementary figures and images for: Divergent effects of short-term and continuous anthropogenic noise exposure on Western Bluebird parental care behavior
Source: PeerJ. 2024 Nov 26;12:e18558. doi: 10.7717/peerj.18558 (PMC11606327; doi:10.7717/peerj.18558)

Amplitude (dB)

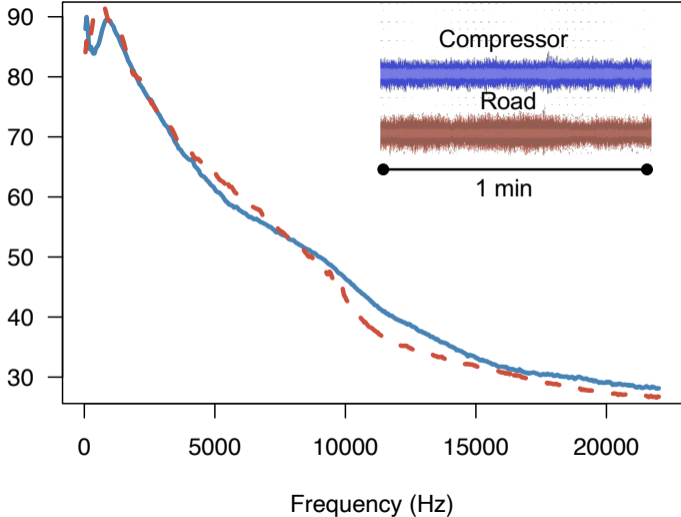

Supplement: Supplemental Information 2 — Power spectra illustrating similar energy across frequencies for a recording at 80 m from Highway 101 in a rural area of the Central Coast of California (red dashed-line) and a recording made at 100 m from a compressor at one of our sites in New Mexico (solid blue line). Inset waveform illustrates fluctuation in amplitude over a 1-min period of noise from both sources. [file peerj-12-18558-s002.pdf]
